# Supplementary material for: Splice-Junction-Based Mapping of Alternative Isoforms in the Human Proteome
Source: Cell Rep. Author manuscript; Available in PMC 2020 Jan 15. (PMC6961840; doi:10.1016/j.celrep.2019.11.026)
Supplement: 2 [file NIHMS1546469-supplement-2.zip › DF1/PXD010154/Testis/Testis_26_LSM2_ELVGKDVVVELK.pdf]

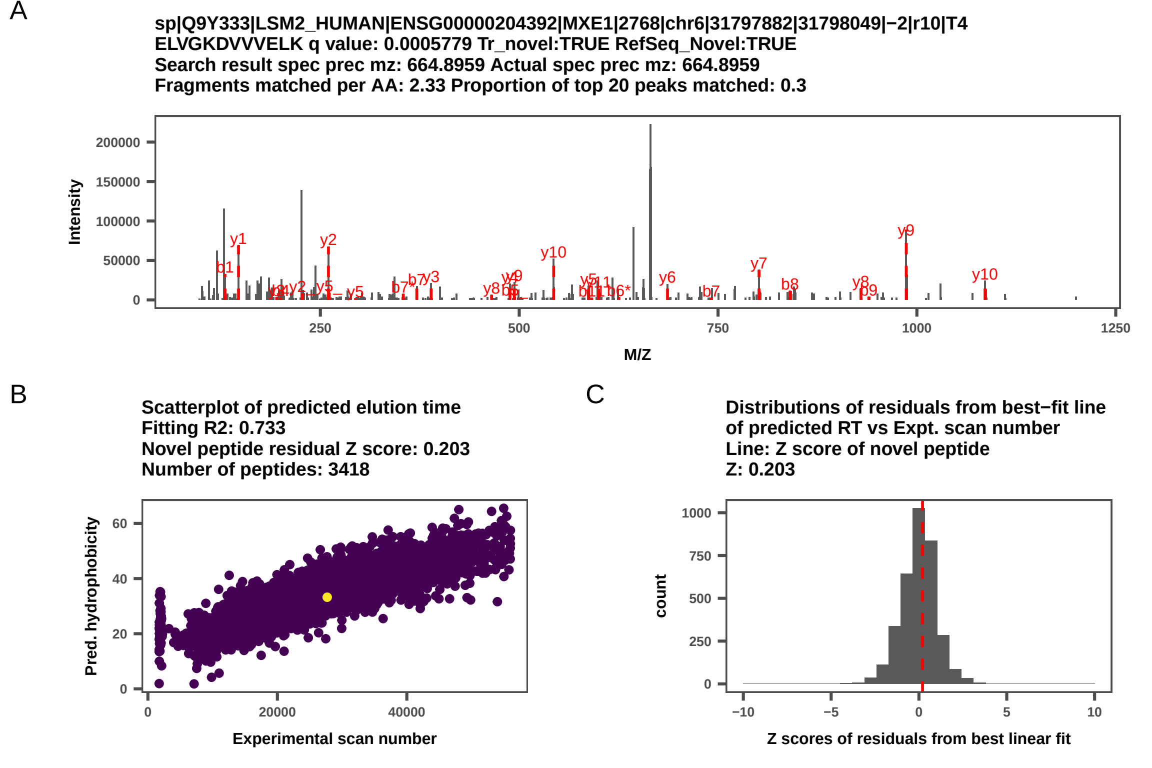
A
sp|Q9Y333|LSM2_HUMAN|ENSG00000204392|MXE1|2768|chr6|31797882|31798049|−2|r10|T4
ELVGKDVVVELK q value: 0.0005779 Tr_novel:TRUE RefSeq_Novel:TRUE
Search result spec prec mz: 664.8959 Actual spec prec mz: 664.8959
Fragments matched per AA: 2.33 Proportion of top 20 peaks matched: 0.3
Intensity
200000
150000
100000
50000
0
M/Z
250
500
750
1000
1250
y1
b1
y2
y10
y6
y7
b8
y9
y10
B
Scatterplot of predicted elution time
Fitting R2: 0.733
Novel peptide residual Z score: 0.203
Number of peptides: 3418
Pred. hydrophobicity
60
40
20
0
0
20000
40000
Experimental scan number
C
Distributions of residuals from best−fit line
of predicted RT vs Expt. scan number
Line: Z score of novel peptide
Z: 0.203
count
1000
750
500
250
0
−10
−5
0
5
10
Z scores of residuals from best linear fit
